# Supplementary material for: The end-joining factor Ku acts in the end-resection of double strand break-free arrested replication forks
Source: Nat Commun. 2017 Dec 7;8:1982. doi: 10.1038/s41467-017-02144-5 (PMC5719404; doi:10.1038/s41467-017-02144-5)
Supplement: Supplementary file 1 — Supplementary Information [file 41467_2017_2144_MOESM1_ESM.docx]

**
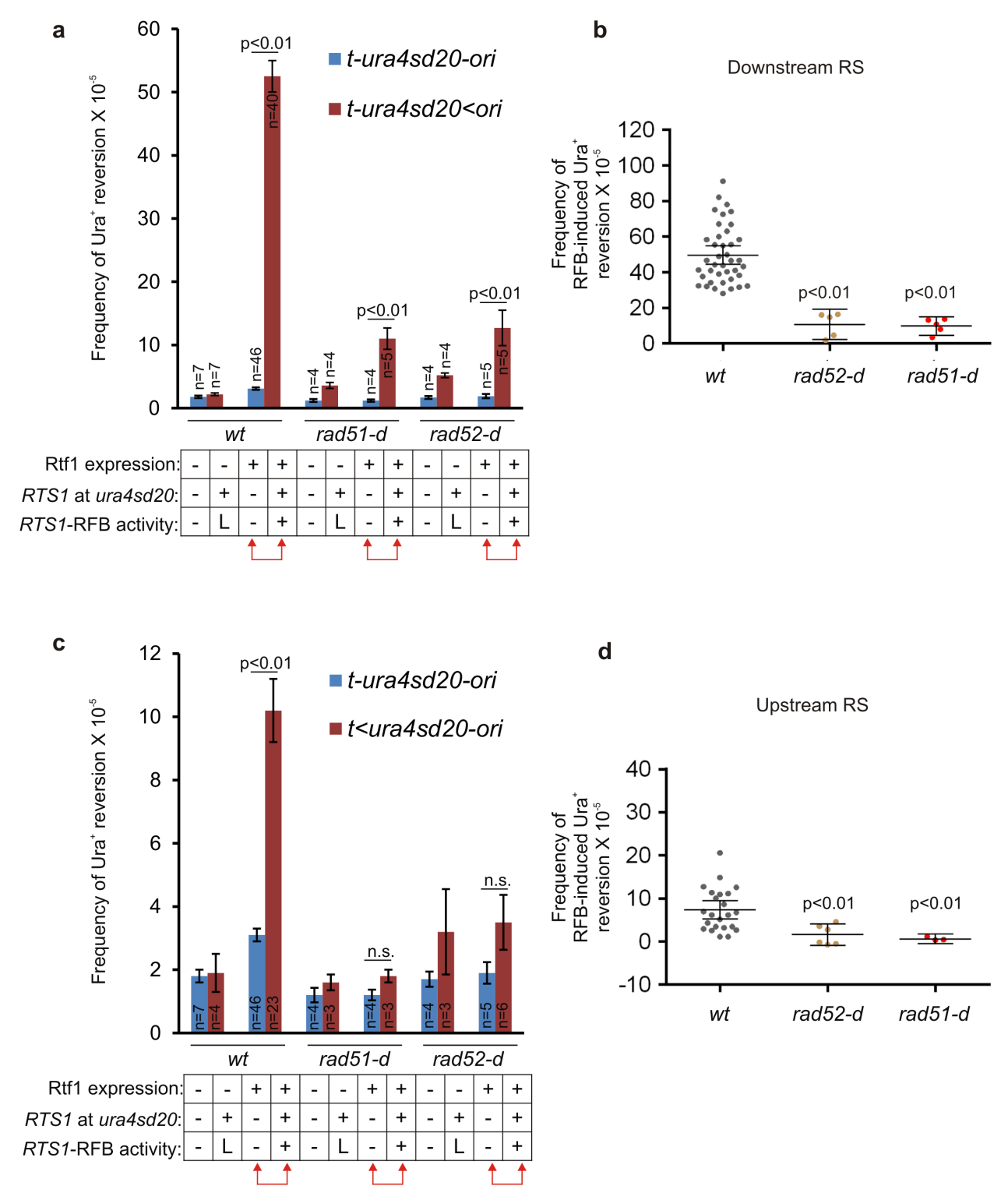
**

**Supplementary Figure 1: A genetic assay to monitor HR-mediated replication restart and investigate fork-resection and the *RTS1*-RFB**

**(a)** Frequency of Ura^+^ reversion in indicated strains, using the indicated constructs containing the *ura4-sd20* reporter gene (*t-ura4sd20-ori* in blue and *t-ura4sd20<ori* in red), in indicated conditions and as previously reported^1^. The *ura4-sd20* allele contains a 20 nt duplication flanked by 5 bp of micro-homology. When the *ura4-sd20* allele is replicated by a restarted fork, the non-processive DNA synthesis undergoes replication slippage (RS) resulting in the deletion of the duplication and the restoration of a functional *ura4*^+^ gene (Ura^+^ reversion)^2^. Values are means of n samples from independent biological replicates ± standard error of the mean (SEM). Statistics were calculated using the Mann and Whitney *U* test. The expression of Rtf1 (“-” for repressed and “+” for expressed), presence of the *RTS1*-RFB (“-” for absence and “+” for presence) and activity of the *RTS1*-RFB (“-” for its absence, “+” for active, and “L” for leaky) are indicated on the panel bellow the histogram. The frequency of downstream RS was increased by 23 fold upon expression of Rtf1 (*t-ura4sd20*<ori, compare Rft1 repressed to expressed situation) and by 17 fold compared to the strain devoid of RFB (*t-ura4sd20-ori*). In *rad51* or *rad52* deleted strains, the leakiness of Rft1 repression was more obvious as the RS frequency was slightly higher when Rft1 was repressed compared to the strain devoid of RFB (compare Rtf1 expression: -, *RTS1*: -, RFB activity: - with Rtf1 expression: -, *RTS1*: +, RFB: L). Thus, to obtain the true occurrence of Ura^+^ reversion by the *RTS1*-RFB, independently of the genetic background, we subtracted the RS frequency of the strain devoid of RFB from the frequency of the strain containing *the t-ura4sd20<ori* construct, upon expression of Rtf1 (red arrows). The red arrows indicate the values used to obtain RFB-induced RS represented on the panel B.

**(b)** Frequency of RFB-Induced Ura^+^ reversion in indicated strains. The frequency of RS monitored with the *t-ura4sd20-ori* construct was subtracted to the frequency obtained with the *t-ura4sd<ori* construct upon expression of Rtf1 to disregard genetic backgrounds effect and obtain the true induction of RS by the *RTS1*-RFB. Each dot represents one value obtained from the subtraction of each value obtained with the *t-ura4sd20<ori* construct to the mean of the values obtained with *t-ura4sd20-ori*. Bars indicate the mean value ± 95 % confidence interval (CI). P-values were calculated using the Mann and Whitney *U* test.

**(c) and (d)** Same representation as (A) and (B), respectively, using the constructs containing the *ura4-sd20* reporter gene (*t-ura4sd20-ori* in blue and *t<ura4sd20-ori* in red), in indicated conditions.


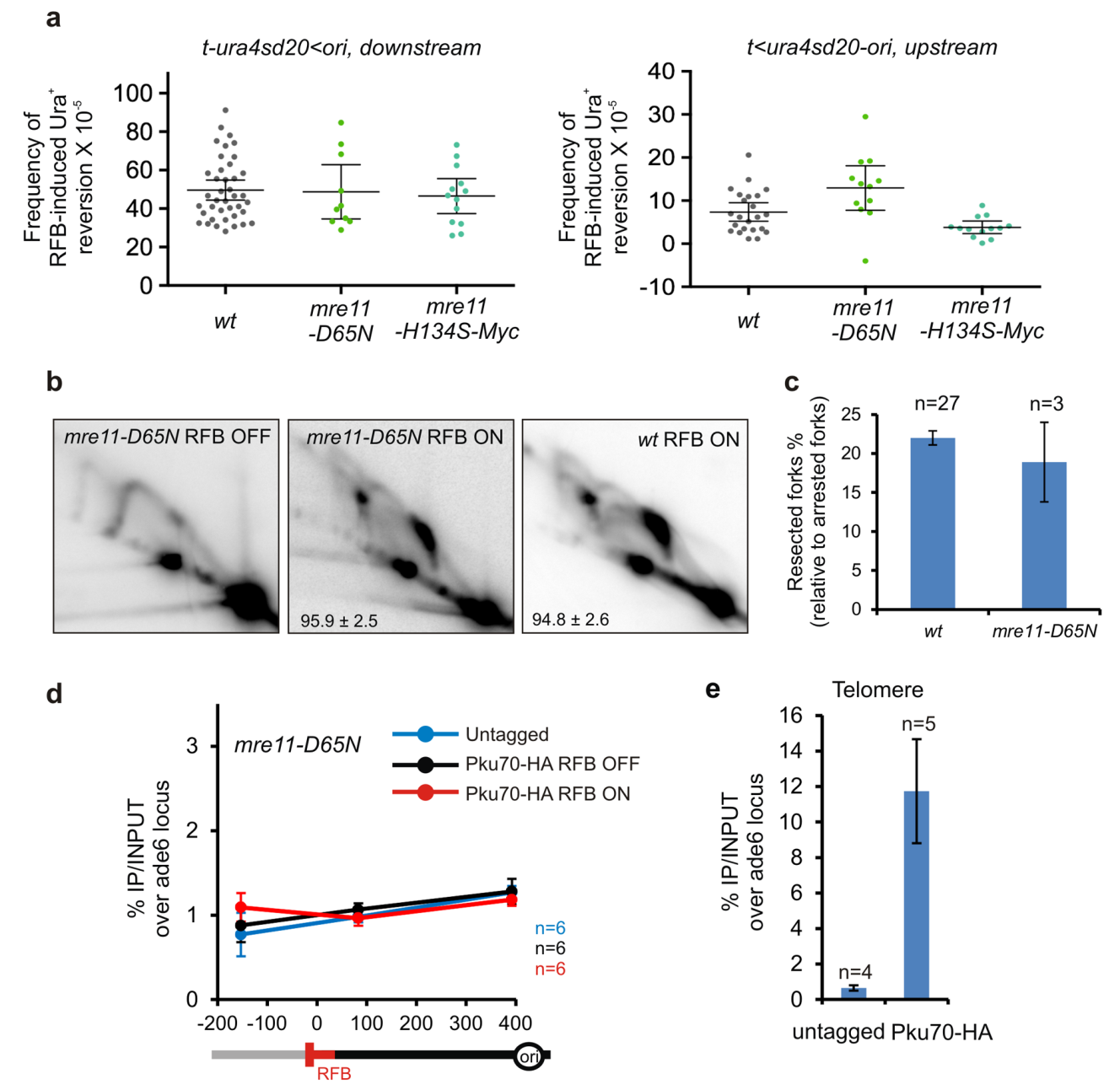


**Supplementary Figure 2: The nuclease activity of Mre11 is dispensable to promote fork-resection and restart**

**(a)** Frequency of downstream RFB-induced Ura^+^ reversion (left panel, *t-ura4sd20<ori*) and upstream RFB-induced Ura^+^ reversion (right panel, *t<ura4sd20-ori*) in indicated strains. Each dot represents one sample from independent biological replicates. Bars indicate the mean value ± 95 % CI. Statistics were calculated using the non-parametric Mann and Whitney *U* test. No statistical differences were detected between *wt* and the two *mre11* nuclease dead alleles.

**(b)** Representative RI analysis by 2DGE in indicated strains upon activation (RFB ON) or not (RFB OFF) of the *RTS1*-RFB. Numbers indicate the % of forks blocked at the *RTS1*-RFB ± SD.

**(c)** Quantification of forks undergoing resection (“tail signal”), relative to the intensity of terminally-arrested forks. Values are means of n samples from independent biological replicates ± 95% CI.

**(d)** Analysis of Ku recruitment to the *RTS1*-RFB by ChIP-qPCR in *mre11-D65N* strain. Upstream and downstream distances from the RFB are indicated in base pairs (bp). Values are means of n samples from independent biological replicates ± SEM.

**(e)** Analysis of Ku recruitment to telomeres in *mre11-D65N* strain. Values are means of n samples from independent biological replicates ± SD.


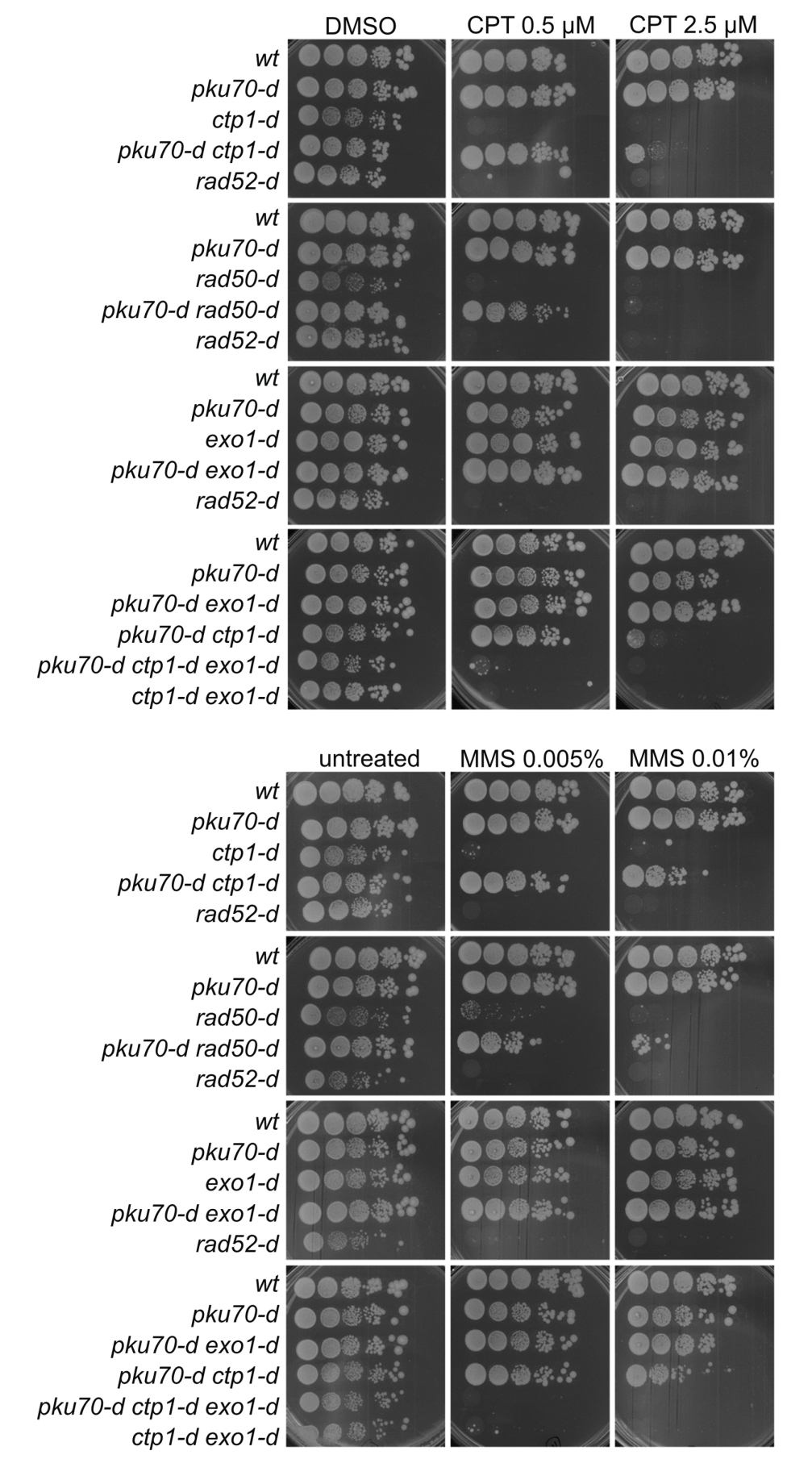


**Supplementary Figure 3: The deletion of *pku70* partially rescues the sensitivity of *ctp1-d* and *rad50-d* cells to CPT and MMS, in an Exo1-dependent manner**

Tenfold serial dilution of indicated strains on plates containing the indicated doses of CPT and MMS.

**
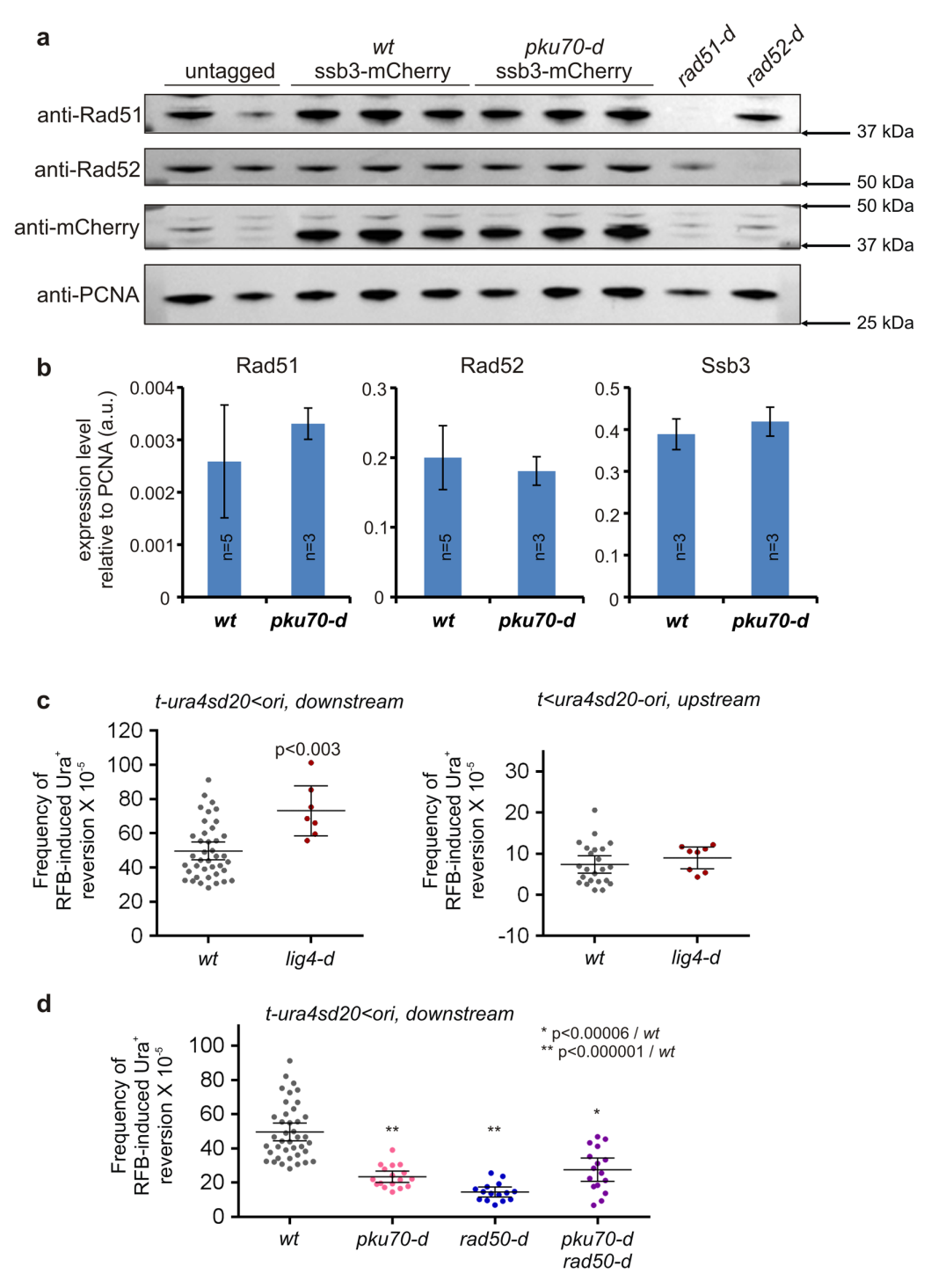
**

**Supplementary Figure 4: The lack of Ku impairs the dynamics of HR-mediated fork restart**, **independently of NHEJ**

**(a)** Expression of Rad51, Rad52 and Ssb3-mCherry in *wt* and *pku70-d* strains. Untagged, *rad51-d* and *rad52-d* strains were included as controls for antibodies specificity. PCNA was used as loading control.

**(b)** Quantification of protein expression relative to PCNA. Values indicate the mean (a.u.) of at least three independent experiments ± SD.

**(c)** Frequency of downstream RFB-induced Ura^+^ reversion (left panel, *t-ura4sd20<ori*) and upstream RFB-induced Ura^+^ reversion (right panel, *t<ura4sd20-ori*) in indicated strains. Each dot represents one sample from independent biological replicates. Bars indicate the mean value ± 95 % CI. Statistics were calculated using the non-parametric Mann and Whitney *U* test.

**(d)** Frequency of downstream RFB-induced Ura^+^ reversion in indicated strains. Each dot represents one sample from independent biological replicates. Bars indicate the mean value ± 95 % CI. Statistics were calculated using the non-parametric Mann and Whitney *U* test.

**
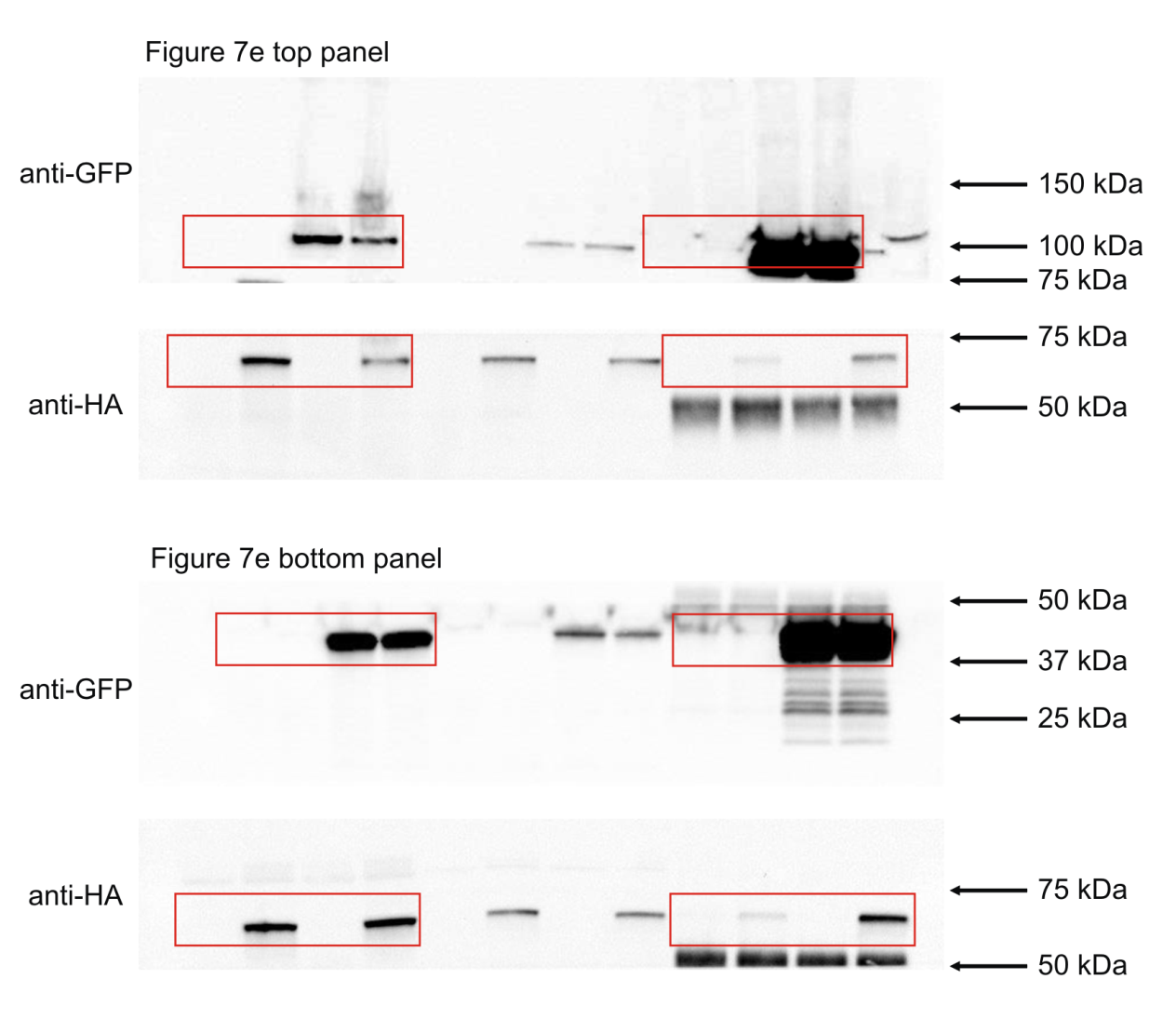
**

**Supplementary Figure 5:** Uncropped blots from figure 7

**Supplementary Table 1**: Strains used in this study (related to all figures)

| Strain |  | *Genotype* | *Reference* |
| --- | --- | --- | --- |
| SL350 | *h-smt0* | *t-ura4^+^<ori sup35:nmt41:rtf1^+^ ade6-704 leu1-32* | *3* |
| YC6 | *h-smt0* | *t-ura4-SD20-ori sup35:nmt41:rtf1^+^ ade6-704 leu1-32* | 2 |
| YC21 | *h-smt0* | *t>ura4-SD20-ori sup35:nmt41:rtf1^+^ ade6-704 leu1-32* | 2 |
| YC13 | *h-smt0* | *t-ura4-SD20<ori sup35:nmt41:rtf1^+^ ade6-704 leu1-32* | 2 |
| YC76 | *h-smt0* | *rad51::KAN t-ura4-SD20-ori sup35:nmt41:rtf1^+^ ade6-704 leu1-32* | 2 |
| YC82 | *h+* | *rad51::KAN t>ura4-SD20-ori sup35:nmt41:rtf1^+^ ade6-704 leu1-32* | *This study* |
| YC80 | *h-smt0* | *rad51::KAN t-ura4-SD20<ori sup35:nmt41:rtf1^+^ ade6-704 leu1-32* | 2 |
| YC86 | *h+* | *rad52::NAT t-ura4-SD20-ori sup35:nmt41:rtf1^+^ ade6-704 leu1-32* | 2 |
| YC91 | *h-smt0* | *rad52::NAT t>ura4-SD20-ori sup35:nmt41:rtf1^+^ ade6-704 leu1-32* | *This study* |
| YC90 | *h-smt0* | *rad52::NAT t-ura4-SD20<ori sup35:nmt41:rtf1^+^ ade6-704 leu1-32* | 2 |
| II264 | *h-* | *exo1::NAT t-ura4-SD20-ori sup35:nmt41:rtf1^+^ ade6-704 leu1-32* | *1* |
| II324 | *h- smt0* | *exo1::NAT t<ura4-SD20-ori sup35:nmt41:rtf1^+^ ade6-704 leu1-32* | *1* |
| II268 | *h-* | *exo1::NAT t-ura4-SD20<ori sup35:nmt41:rtf1^+^ ade6-704 leu1-32* | 2 |
| II383 | *h-* | *rqh1::KAN t-ura4-SD20-ori sup35:nmt41:rtf1^+^ ade6-704 leu1-32* | *This study* |
| II376 | *h-* | *rqh1::KAN t<ura4-SD20-ori sup35:nmt41:rtf1^+^ ade6-704 leu1-32* | *This study* |
| II381 | *h-* | *rqh1::KAN t-ura4-SD20<ori sup35:nmt41:rtf1^+^ ade6-704 leu1-32* | *This study* |
| II487 | *h-* | *rqh1::KAN exo1::NAT t-ura4-SD20-ori sup35:nmt41:rtf1^+^ ade6-704 leu1-32* | *This study* |
| II492 | *h-* | *rqh1::KAN exo1::NAT t<ura4-SD20-ori sup35:nmt41:rtf1^+^ ade6-704 leu1-32* | *This study* |
| II490 | *h-* | *rqh1::KAN exo1::NAT t<ura4-SD20-ori sup35:nmt41:rtf1^+^ ade6-704 leu1-32* | *This study* |
| II445 | *h- smt0* | *ctp1::NAT t-ura4-SD20-ori sup35:nmt41:rtf1^+^ ade6-704 leu1-32* | *This study* |
| II440 | *h- smt0* | *ctp1::NAT t>ura4-SD20-ori sup35:nmt41:rtf1^+^ ade6-704 leu1-32* | *This study* |
| II437 | *h- smt0* | *ctp1::NAT t-ura4SD20<ori sup35:nmt41:rtf1^+^ ade6-704 leu1-32* | *This study* |
| II518 | *h-* | *ctp1::HYGRO t-ura4-SD20-ori sup35:nmt41:rtf1^+^ ade6-704 leu1-32* | *This study* |
| II527 | *h+* | *ctp1::HYGRO t>ura4-SD20-ori sup35:nmt41:rtf1^+^ ade6-704 leu1-32 his^+^* | *This study* |
| II525 | *h-* | *ctp1::HYGRO t-ura4-SD20<ori sup35:nmt41:rtf1^+^ ade6-704 leu1-32 his^+^* | *This study* |
| YC65 | *h^-^smt0* | *rad50::KAN t-ura4-SD20-ori sup35:nmt41:rtf1^+^ ade6-704 leu1-32* | 2 |
| YC70 |  | *rad50::KAN t>ura4-SD20-ori sup35:nmt41:rtf1^+^ ade6-704 leu1-32* | *This study* |
| YC67 | *h^-^smt0* | *rad50::KAN t-ura4-SD20<ori sup35:nmt41:rtf1^+^ ade6-704 leu1-32* | 2 |
| II541 | *h-* | *ctp1::HYGRO rad50::KAN t-ura4-SD20-ori sup35:nmt41:rtf1^+^ ade6-704 leu1-32* | *This study* |
| II547 | *h-* | *ctp1::HYGRO rad50::KAN t>ura4-SD20-ori sup35:nmt41:rtf1^+^ ade6-704 leu1-32* | *This study* |
| II544 | *h-* | *ctp1::HYGRO rad50::KAN t-ura4-SD20<ori sup35:nmt41:rtf1^+^ ade6-704 leu1-32* | *This study* |
| II483 | *h+* | *ctp1::HYGRO exo1::NAT t-ura4-SD20-ori sup35:nmt41:rtf1^+^ ade6-704* | *This study* |
| II486 | *h-* | *ctp1::HYGRO exo1::NAT t>ura4-SD20-ori sup35:nmt41:rtf1^+^ ade6-704* | *This study* |
| II484 | *h-* | *ctp1::HYGRO exo1::NAT t-ura4-SD20<ori sup35:nmt41:rtf1^+^ ade6-704* | *This study* |
| II532 | *h-* | *rad50::KAN exo1::NAT t-ura4-SD20-ori sup35:nmt41:rtf1^+^ ade6-704 leu1-32* | *This study* |
| II535 | *h-* | *rad50::KAN exo1::NAT t-ura4-SD20<ori sup35:nmt41:rtf1^+^ ade6-704 leu1-32* | *This study* |
| II538 | *h-* | *rad50::KAN exo1::NAT t>ura4-SD20-ori sup35:nmt41:rtf1^+^ ade6-704 leu1-32* | *This study* |
| YC259 | *h-* | *mre11-D65N t-ura4-SD20-ori sup35:nmt41:rtf1^+^ ade6-704 leu1-32* | *This study* |
| YC264 | *h-* | *mre11-D65N t>ura4SD20-ori sup35:nmt41:rtf1^+^ ade6-704 leu1-32* | *This study* |
| YC262 | *h-* | *mre11-D65N t-ura4-SD20<ori sup35:nmt41:rtf1^+^ ade6-704 leu1-32* | *This study* |
| II594 | *h+* | *pku70::leu2 t-ura4-SD20-ori sup35:nmt41:rtf1^+^ ade6-704 leu1-32* | *This study* |
| II603 | *h-* | *pku70::leu2 t>ura4-SD20-ori sup35:nmt41:rtf1^+^ ade6-704 leu1-32* | *This study* |
| II598 | *h+* | *pku70::leu2 t-ura4-SD20<ori sup35:nmt41:rtf1^+^ ade6-704 leu1-32* | *This study* |
| AS18 | *h+* | *pku70::leu2 ctp1::HYGRO t-ura4-SD20-ori sup35:nmt41:rtf1^+^ ade6-704 leu1-32* | *This study* |
| AS30 | *h-* | *pku70::leu2 ctp1::HYGRO t>ura4-SD20-ori sup35:nmt41:rtf1^+^ ade6-704 leu1-32* | *This study* |
| AS23 | *h-* | *pku70::leu2 ctp1::HYGRO t-ura4S-D20<ori sup35:nmt41:rtf1^+^ ade6-704 leu1-32* | *This study* |
| AS1 | *h-* | *pku70::leu2 rad50::KAN t-ura4-SD20-ori sup35:nmt41:rtf1^+^ ade6-704 leu1-32* | *This study* |
| AS6 | *h-* | *pku70::leu2 rad50::KAN t>ura4SD20-ori sup35:nmt41:rtf1^+^ ade6-704 leu1-32* | *This study* |
| AS12 | *h+* | *pku70::leu2 rad50::KAN t-ura4-SD20<ori sup35:nmt41:rtf1^+^ ade6-704 leu1-32* | *This study* |
| AS52 | *h-* | *pku70::leu2 exo1 ::NAT t-ura4-SD20-ori sup35:nmt41:rtf1^+^ ade6-704 leu1-32* | *This study* |
| AS56 | *h+* | *pku70::leu2 exo1 ::NAT t>ura4-SD20-ori sup35:nmt41:rtf1^+^ ade6-704 leu1-32* | *This study* |
| AS178 | *h+* | *pku70::leu2 exo1 ::NAT t-13xter-ura4-SD20-ura5<ori sup35:nmt41:rtf1^+^ ura5::HYGRO ade6-704 leu1-32* | *This study* |
| AS63 | *h+* | *pku70::leu2 exo1 ::NAT ctp1::HYGRO t-ura4-SD20-ori sup35:nmt41:rtf1^+^ ade6-704 leu1-32* | *This study* |
| AS67 | *h-* | *pku70::leu2 exo1 ::NAT ctp1::HYGRO t>ura4sd20-ori sup35:nmt41:rtf1^+^ ade6-704 leu1-32* | *This study* |
| AS70 | *h-* | *pku70::leu2 exo1 ::NAT ctp1::HYGRO t-ura4-SD20<ori sup35:nmt41:rtf1^+^ ade6-704 leu1-32* | *This study* |
| AS120 | *h+* | *lig4::KAN t-ura4-SD20-ori sup35:nmt41:rtf1^+^ ade6-704 leu1-32* | *This study* |
| AS116 | *h+* | *lig4::KAN t>ura4sd20-ori sup35:nmt41:rtf1^+^ ade6-704 leu1-32* | *This study* |
| AS111 | *h+* | *lig4::KAN t-ura4-SD20<ori sup35:nmt41:rtf1^+^ ade6-704 leu1-32 his3-DL* | *This study* |
| YC266 | *h-* | *t-13xter-ura4-SD20-ura5<ori sup35:nmt41:rtf1^+^ ura5::HYGRO ade6-704 leu1-32* | 4 |
| AS128 | *h-* | *pKu70::leu2 t-13xter-ura4-SD20-ura5<ori sup35:nmt41:rtf1^+^ ura5 ::HYGRO ade6-704 leu1-32* | *This study* |
| II606 | *h-* | *ctp1::NAT t-13xter-ura4-SD20-ura5<ori sup35:nmt41:rtf1^+^ ura5::HYGRO ade6-704 leu1-32* | *This study* |
| AS182 | *h+* | *pku70 :: leu1 ctp1::NAT t-13xter-ura4-SD20-ura5<ori sup35:nmt41:rtf1^+^ ura5::HYGRO ade6-704 leu1-32* | *This study* |
| II561 | *h-* | *rad50::KAN t-13xter-ura4-SD20-ura5<ori sup35:nmt41:rtf1^+^ ura5::HYGRO ade6-704 leu1-32* | *This study* |
| AS254 | *h+* | *pku70::leu2 rad50 ::KAN t-13xter-ura4-SD20-ura5<ori sup35:nmt41:rtf1^+^ ura5 ::HYGRO ade6-704 leu1-32* | *This study* |
| AS97 | *h90* | *pku70-3HA:ura4^+^ t-ura4-SD20<ori sup35:nmt41:rtf1^+^ ade6-704 leu1-32* | *This study* |
| AS132 | *h+* | *rad50::KAN pku70-3HA:ura4^+^ t-ura4-SD20<ori sup35:nmt41:rtf1^+^ ade6-704 leu1-32* | *This study* |
| AS134 | *h+* | *ctp1::HYGRO pku70-3HA:ura4^+^ t-ura4-SD20<ori sup35:nmt41:rtf1^+^ ade6-704 leu1-32* | *This study* |
| AS359 | *h-* | *mre11-D65N pku70-3HA:ura4^+^ t-ura4S-D20<ori sup35:nmt41:rtf1^+^ ade6-704 leu1-32* | *This study* |
| AS39 | *h+* | *arg3::psv40-GFP-LacI** ssb3-mCherry:KAN lacO 7,9Kb:KAN t-ura4^+^<ori sup35:nmt41:rtf1^+^ ade6-704 leu1-32* | 4 |
| AS206 | *h+* | *pku70::leu2 arg3::psv40-GFP-LacI** ssb3-mCherry:KAN lacO 7,9Kb:KAN t-ura4^+^<ori sup35:nmt41:rtf1^+^ ade6-704 leu1-32* | *This study* |
| AC147 | *h+* | *ssb3-YFP:NAT t-ura4^+^<ori sup35:nmt41:rtf1^+^ ade6-704 leu1-32* | *This study* |
| SL1507 | *h+* | *pku70-3HA:ura4^+^ ssb3-YFP:NAT t-ura4-SD20-ori sup35:nmt41:rtf1^+^ ade6-704 leu1-32* | *This study* |
| AC61 | *h-smt0* | *rad11-YFP:NAT t-ura4^+^<ori sup35:nmt41:rtf1^+^ ade6-704 leu1-32* | *This study* |
| SL1498 | *h+* | *pku70-3HA:ura4^+^ rad11-YFP:NAT t-ura4-SD20<ori sup35:nmt41:rtf1^+^ ade6-704 leu1-32* | *This study* |
| AS344 | *h+* | *mre11-H134S-13*myc-KAN t-ura4-SD20-ori sup35:nmt41:rtf1^+^ ade6-704 leu1-32* | *This study* |
| AS345 | *h-* | *mre11-H134S-13*myc-KAN t-ura4-SD20<ori sup35:nmt41:rtf1^+^ ade6-704 leu1-32* | *This study* |
| AS347 | *h-* | *mre11-H134S-13*myc-KAN t<ura4-SD20-ori sup35:nmt41:rtf1^+^ ade6-704 leu1-32* | *This study* |

**Supplementary Table 2:** List of primers used in this study (related to Fig. 1g, 2d, 4a, 5d and 7d)

| **Name** | **Distance (bp) from RTS1 / position** | **Sequence (5’-3’)** | **Experiment** |
| --- | --- | --- | --- |
| L3F | 110 | TTTAAATCAAATCTTCCATGCG | ssDNA qPCR |
| L3R |  | TGTACCCATGAGCAAACTGC | ssDNA qPCR |
| L400F | 450 | ATCTGACATGGCATTCCTCA | ssDNA qPCR |
| L400R |  | GATGCCAGACCGTAATGACA | ssDNA qPCR |
| L1800F | 1800 | GGCAAAGTAGATCCGACAGC | ssDNA qPCR |
| L1800R |  | TGAATACGCCGTTACTCCTAAAG | ssDNA qPCR |
| L2200F | 2200 | AAGGCAAGAAACGCTGAGAC | ssDNA qPCR |
| L2200R |  | GGCATGCATACTACCCGATAA | ssDNA qPCR |
| II50F | Locus control  (ChrII) | CACCGCAGTTCTACGTATCCT | ssDNA qPCR |
| II50R |  | CGATGTAACGGTATGCGGTA | ssDNA qPCR |
| L5F | -153 | AGGGCATTAAGGCTTATTTACAGA | ChIP Pku70-HA, Rad51 and Ssb3-YFP |
| L5R |  | TCACGTTTAATTTCAAACATCCA | ChIP Pku70-HA, Rad51 and Ssb3-YFP |
| L3F | 110 | TTTAAATCAAATCTTCCATGCG | ChIP Pku70-HA, Rad51 and Ssb3-YFP |
| L3R |  | TGTACCCATGAGCAAACTGC | ChIP Pku70-HA, Rad51 and Ssb3-YFP |
| L400F | 450 | ATCTGACATGGCATTCCTCA | ChIP Pku70-HA, Rad51 and Ssb3-YFP |
| L400R |  | GATGCCAGACCGTAATGACA | ChIP Pku70-HA, Rad51 and Ssb3-YFP |
| Ade6-23 | Chromosome  III | GGCTGCCTCTACCATCATTC | ChIP Pku70-HA, Rad51 and Ssb3-YFP |
| Ade6-25 |  | TTAAGCTGAGCTGCCAAGGT | ChIP Pku70-HA, Rad51 and Ssb3-YFP |
| TelF^5^ | Telomeres | CGGCTGACGGGTGGGGCCCAATA | ChIP Pku70-HA |
| TelR^5^ |  | GTGTGGAATTGAGTATGGTGAA | ChIP Pku70-HA |

Supplementary References

1. Tsang, E. *et al.* The extent of error-prone replication restart by homologous recombination is controlled by Exo1 and checkpoint proteins. *J. Cell Sci.* **127,** 2983–2994 (2014).

2. Iraqui, I. *et al.* Recovery of arrested replication forks by homologous recombination is error-prone. *PLoS Genet.* **8,** (2012).

3. Lambert, S., Watson, A., Sheedy, D. M., Martin, B. & Carr, A. M. Gross chromosomal rearrangements and elevated recombination at an inducible site-specific replication fork barrier. *Cell* **121,** 689–702 (2005).

4. Ait Saada, A. *et al.* Unprotected Replication Forks Are Converted into Mitotic Sister Chromatid Bridges. *Mol. Cell* **66,** 398–410.e4 (2017).

5. Tomita, K. *et al.* Competition between the Rad50 Complex and the Ku Heterodimer Reveals a Role for Exo1 in Processing Double-Strand Breaks but Not Telomeres. **23,** 5186–5197 (2003).
