# Supplementary material for: The end-joining factor Ku acts in the end-resection of double strand break-free arrested replication forks
Source: Nat Commun. 2017 Dec 7;8:1982. doi: 10.1038/s41467-017-02144-5 (PMC5719404; doi:10.1038/s41467-017-02144-5)
Supplement: Supplementary file 3 — Description of Additional Supplementary Files [file 41467_2017_2144_MOESM3_ESM.docx]

**Description of Additional Supplementary Files**

File Name: Supplementary Data 1

Description: Statistics on RFB-induced Ura+ reversion (related to Fig 1c, 2a, 3a, 4a, 5a).
